# Bevacizumab in Glaucoma: Where do We Stand?

**DOI:** 10.5005/jp-journals-10008-1110

**Published:** 2012-08-16

**Authors:** Anjani Khanna

**Affiliations:** Glaucoma Services, Department of Ophthalmology, Government Medical College and Hospital, Chandigarh, India

**Keywords:** Bevacizumab, Glaucoma, Intraocular pressure, VEGF.

## Abstract

The use of antivascular endothelial growth factors such as bevacizumab and ranibizumab has brought about a revolution in management protocols of various ophthalmic disorders. A lot has been written about these agents, still lacunae exist in our understanding due to paucity of randomized control trials with large number of patients. This brief review attempts to throw light on the clinical applications of these molecules for glaucoma.

**How to cite this article:**

Khanna A. Bevacizumab in Glaucoma: Where do We Stand? J Current Glau Prac 2012;6(2):75-78.

## CONCEPT

The concept of a diffusable factor that affects the budding of new vessels was proposed initially in 1954,^1^ which has since been substantiated by the active research into the signal transduction pathway that leads to ocular angiogenesis.

A critically important angiogenic factor in ocular neovascularization is VEGF―vascular endothelial growth factor, an endothelial cell mitogen; which till date is the best studied and described component of this pathway. VEGF is a 46 kDa glycoprotein noted in highly vascularized tumors.^2^ Four isoforms have been identified and are instrumental in both the normal and pathologic process of angiogenesis and furthermore―as a vascular permeability factor inducing hyperpermeability, endothelial cell migration and proliferation.^2-4^ The critical role of VEGF in the mediation of active anterior segment intraocular neovascularization in patients with ischemic retinal disease has been demonstrated through observations of significantly increased levels of VEGF in the vitreous and aqueous humor.^5-10^

### Molecule

Bevacizumab is a recombinant humanized anti-VEGF immunoglobulin G1 (IgG1) approved as an antiangiogenic agent for the treatment of metastatic colorectal cancer in combination with chemotherapy.^6^ By binding to two receptor kinases [VEGFR-1 (Flt-1) and VEGFR-2 (KDR, Flk-1)], bevacizumab is able to downregulate the mitogenic, angiogenic and permeability-enhancing effects of VEGF A. Avastin^®^ (bevacizumab) is clear to slightly opalescent, colorless to pale brown, sterile solution with pH 6.2. It was originally designed for intravenous (IV) infusion and is supplied in 100 mg and 400 mg preservative-free, single-use vials to deliver 4 ml or 16 ml of Avastin^®^ (25 mg/ml). The product is formulated in alpha-trehalose dihydrate, sodium phosphate (monobasic, monohydrate), sodium phosphate (dibasic, anhydrous), polysorbate and water for injection.

### Clinical Applications for Glaucoma

Boon for Neovascular Glaucoma

Neovascular glaucoma (NVG) occurs secondary to pathologies that lead to retinal ischemia and is associated with poor visual outcome.^7,8^ Iris and angle neovascu-larization in the late stages can lead to the development of peripheral anterior synechiae that occludes the angle and results in elevated intraocular pressure (IOP) that does not respond to traditional topical glaucoma therapy. Multiple panretinal photocoagulation sittings have been shown to downregulate the release of VEGF into the vitreous cavity.^9^ Media opacities, such as a mature cataract or vitreous hemorrhage, may preclude laser in some cases. Cyclophoto-coagulation or invasive surgery is often required to reduce IOP.

VEGF, a potent mitogen specific for vascular endothelial cells, is upregulated under conditions of retinal ischemia and NVG. A downregulation in its production through the use of VEGF inhibitors would logically stifle the neovascularization. Several case series highlight the regression of neovascularization of both the iris and angle with the use of VEGF inhibitors.^9-17^

Sasamoto et al^18^ found significant correlation of aqueous humor VEGF levels with IOP and doses of 0.1 mg and 1.0 mg intravitreal bevacizumab (IVB) to be effective for treating NVG within at least 6 months after the initial injection.

Iliev et al^15^ described marked regression of anterior segment neovascularization and relief of symptoms in the first 2 days after the use of IVB (intravitreal bevacizumab), 1.25 mg/0.05 ml, for refractory NVG.

With the use of iris fluorescein angiography, Grisanti et al^18^ studied the effects of IVB on NVI. They presented a case series of six eyes in three patients who had NVG and NVI due to CRVO or PDR and received 1.0 mg of IVB. They noted a decrease in iris fluorescein angiography leakage as early as 1 day after injection. Topical medications did not control IOP in the patients who already had developed peripheral anterior synechiae.^19^

Bakri et al studied the pharmacokinetics of bevacizumab and found that the vitreous half-life of 1.25 mg IVB is 4.32 days in a rabbit eye.^20^ They also found small amounts of bevacizumab in the serum and in the fellow uninjected eye.

Wakabayashi et al concluded in their study that IVB effectively stabilized NVI activity and controlled IOP in patients with NVI alone.^21^ But, in patients with advanced NVG and closed angles, IVB did not control IOP. It did, however, show promise as an adjunct to improve subsequent surgeries.

Recently, the effect of bevacizumab to posterior chamber behind iris (BIPC) combined with seton implantation in NVG patients has been studied and the researchers found significant reduction of iris neovascularization within 1 week after the injection and the effect persisted for 6 months.^22^ During the drainage device implantation, they noted decreased peroperative risk of anterior segment bleeding, increased surgical comfort and prevention of failure of filtration procedure by inhibition of reproliferation.

Marey et al^23^ combined IVB with retinal photocoagulation in 20 eyes with NVG, nine of which underwent subsequent subscleral trabeculectomy with mitomycin C (MMC). All cases showed complete regression of iris neovessels at 2 months postinjection with recurrence of iris neovessels in four cases (20%) at 4 months and in 14 cases (70%) at 8 months follow-up. The mean IOP dropped significantly from 41.45 ± 5.89 mm Hg preoperatively to 19.3 ± 5.5 mm Hg and 17.75 ± 3.74 mm Hg at 6 and 12 months postoperatively, respectively. The success rate of subscleral trabeculectomy with mitomycin C after intravitreal bevacizumab was good at 77.8%. They concluded that intravitreal bevacizumab has a role in regression of iris neovessels and IOP control in neovascular glaucoma and increases the success rate of subscleral trabeculectomy with MMC; but this role had a limited time and reinjection is needed to maintain the effect.

Results for anti-VEGF agents for NVG seem promising in retarding and reversing the growth of vessels in the angle. In absence of peripheral anterior synechiae, bevacizumab has profound effects on IOP control. Patients experience less ocular discomfort soon after its use. In conjunction with Panretinal photocoagulation (PRP), the use of anti-VEGF agents holds great promise in improving the otherwise dismal long-term visual prognosis for patients with NVG.

### Is Bevacizumab Meant only for Regression of Neovascularization?

Cornish et al^24^ postulated that in addition to bevacizumab’s antineovascular effect, it modulates wound healing, thereby allowing the trabeculectomy to potentially be more successful in this often difficult to manage subgroup.

Yoshida et al^25^ demonstrated reduced vascular permeability, decreased inflammatory reaction, loss of vascular function and endothelial cell degeneration in trabecular tissues of patients undergoing trabeculectomy for NVG.

Due to anti-VEGF’s effect on wound healing, it has been studied as an adjunct in trabeculectomy surgery as well.

### Wound Modulation in Trabeculectomy

The predictability in the outcome of trabeculectomy is hampered by variability in the wound healing response. Tenon fibroblasts are the main effector cells in the initiation and mediation of most wound healing and fibrotic scar formation.

After trabeculectomy and other forms of glaucoma filtration surgery, the conjunctival and episcleral fibrosis occurs as a result of progressive fibroblast migration, proliferation, collagen deposition and angiogenesis at the site of filtration. Histologic studies have shown that the maximum proliferation of subconjunctival fibroblasts occurs in the third to fifth postoperative day. Pharmacologic enhancement of trabeculectomy using 5-fluorouracil (5-FU) and MMC has improved rates of success significantly. However, the nonspecific mechanism of action of these agents may result in widespread cell death and thin-walled avascular blebs that are susceptible to leakage, infection and dysesthesias.^26^

VEGF seem to drive fibrosis via angiogenesis, but also there is evidence of a more direct effect on fibroblastic activity. VEGF has been shown to be a mediator in the signal transduction cascade leading to fibroblast migration and proliferation. As a powerful inducer of angiogenesis, VEGF also promotes early migration of inflammatory cells and fibroblasts.

Studies by Bergen et al^27^ on various isoforms of VEGF show VEGF165 and VEGF121 predominantly affect blood vessel growth, whereas VEGF189 is more important in fibrosis. Selective inhibition of VEGF165 by pegaptinib (Macugen, Pfizer, an aptamer which specifically blocks VEGF165) may, therefore, be less effective to reduce ocular scar formation than nonselective VEGF inhibition by bevacizumab.

In the first report describing the use of bevacizumab to modulate wound healing in humans, Kahook et al noted a significant and lasting decrease in IOP after a bleb needling procedure after failed trabeculectomy.^28^

The utility of subconjunctival bevacizumab injections administered proximal to blebs after trabeculectomy at the earliest sign of vascularization was suggested by Kapetansky et al.^29^ Nearly two thirds of the blebs had an observable reduction in vascularity in addition to an IOP drop 1 month after injection. Improved results were noted when the injections were given earlier in the postoperative phase.

Grewal and colleagues gave subconjunctival injection of bevacizumab (1.25 mg/0.05 ml) adjacent to the bleb at time of trabeculectomy in 12 patients.^30^ The mean preoperative IOP was 24.4 mm Hg and decreased to a mean of 11.6 mm Hg at 6 months. They concluded that subconjunctival bevacizumab is a potential adjunctive treatment for reducing the incidence of bleb failure after trabeculectomy. An interesting observation in their study was the increasing bleb vascularity that occurred at month 3, which they stated might decrease the incidence of thin cystic blebs that frequently develop after MMC-augmented trabeculectomy surgery.

Nilforushan et al^31^ compared the outcome of trabeculectomy with subconjunctival bevacizumab with trabeculectomy with MMC in 36 eyes and found no significant difference in bleb morphologic features between the two groups, IOP reduced significantly in both the groups, reduction being more prominent in MMC group and concluded bevacizumab to be safe and effective in terms of IOP control.

In contrast, Sedghipour et al^32^ found no effect of subconjunctival bevacizumab (0.2 mg) on IOP control after trabeculectomy compared to placebo in primary open angle glaucoma patients.

### Adverse Events associated with Bevacizumab

As the use of bevacizumab in the treatment of various ocular pathologies has emerged, the short-term safety data for intravitreal administration suggest the adverse event rate to not exceed 0.21%, with a majority of these events related to the administration of any intravitreal injection.

Higashide et al^33^ in their retrospective study of 84 eyes with NVG found that 3% cases with NVG due to ocular ischemic syndrome developed central retinal artery occlusion. None of the cases had marked inflammation, lens injuries, marked vitreous hemorrhage, retinal detachment, endophthalmitis and none experienced systemic side-effects, including myocardial infarction and cerebrovascular accidents within 3 months after bevacizumab injection.

Wittstrom et al^34^ investigated the electrophysiologic effect of intravitreal bevacizumab and concluded that it may reduce the photoreceptor function in NVG patients.

Safety data from long-term randomized controlled trials for bevacizumab is still lacking. In a randomized, prospective interventional study by Chua et al, 43 eyes of 39 patients who underwent uncomplicated primary antimetabolite augmented trabeculectomy who subsequently required postoperative subconjunctival 5-FU injection within 4 weeks of surgery were recruited; 21 eyes were randomized to subconjunctival injections of 5-FU and 22 eyes to combined 5-FU/bevacizumab.^35^ By 18 months after receiving the injections, 47.4% of the 5-FU/bevacizumab group exhibited central bleb avascularity compared with 21.1% of the 5-FU group (Fisher’s exact test, p = 0.17). Two bleb complications (1 blebitis; 1 suture abscess) were noted in the 5-FU/bevacizumab group. The authors suggested the need for a larger clinical trial to further evaluate the safety and efficacy of bevacizumab as a modulating agent in glaucoma filtration surgery.

### Take Home Message

The antiangiogenic and antifibroblastic properties of the recently introduced anti-VEGF agents have led to their early adoption in treating NVG and influencing wound modulation posttrabeculectomy. Prospective multicenter studies are still lacking for these pharmacotherapies for determining treatment regimens, most appropriate route of delivery, optimum dose for each agent, adverse effects and potential patient population that might be more susceptible to currently unknown side effects. In addition, the potential for anti-VEGF delivery systems used in conjunction with glaucoma drainage devices may be explored.
